# Clinical utility and future perspectives of liquid biopsy in colorectal cancer

**DOI:** 10.1038/s43856-025-00852-4

**Published:** 2025-04-24

**Authors:** Giorgio Patelli, Luca Lazzari, Giovanni Crisafulli, Andrea Sartore-Bianchi, Alberto Bardelli, Salvatore Siena, Silvia Marsoni

**Affiliations:** 1 IFOM ETS—The AIRC Institute of Molecular Oncology, Milan, Italy; 2 Department of Oncology and Hemato-Oncology, University of Milan, Milan, Italy; 3 Niguarda Cancer Center, Department of Hematology, Oncology, and Molecular Medicine, Grande Ospedale Metropolitano Niguarda, Milan, Italy; 4 Division of Clinical Research and Innovation, Grande Ospedale Metropolitano Niguarda, Milan, Italy; 5 Department of Oncology, Molecular Biotechnology Center, University of Torino, Turin, Italy

**Keywords:** Tumour biomarkers, Predictive markers, Colorectal cancer

## Abstract

Patelli et al. critically evaluate the clinical utility of liquid biopsy-based circulating tumor DNA (ctDNA) analyses in colorectal cancer management. By addressing its applications across metastatic, locoregional, and early disease settings, the authors highlight both the transformative potential and current limitations of ctDNA in guiding personalized treatment decisions.

## Teaser

Liquid biopsy analyses of circulating tumor DNA are emerging as transformative tools for colorectal cancer management, enabling disease detection, monitoring, and treatment. Here, we critically assess its evidence-based utility in current clinical practice, seeking to align physicians’ and patients’ expectations.

## Introduction

**Fig. 1:**
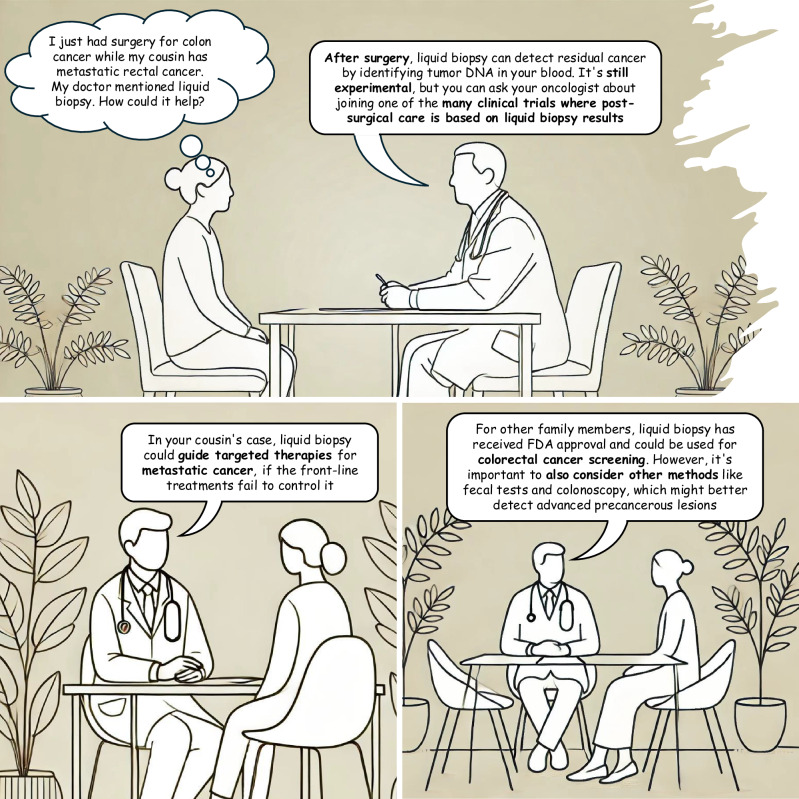
Clinical applications of ctDNA tests in CRC. ctDNA Circulating Tumor DNA. Background image was generated using DALL·E (openai.com) under OpenAI’s content policy, which permits use and publication. The final figure was edited using standard graphic tools.

Colorectal cancer (CRC) management has transformed with the growing integration of liquid biopsy in everyday clinical practice. Liquid biopsy, a non-invasive technique that detects tumor DNA released into the bloodstream (ctDNA), offers a convenient method to characterize cancer via routine blood draws^1^. ctDNA shows potential for cancer detection, monitoring, and therapeutic decision-making^1^. Given the increasing popularity of ctDNA tests and availability of direct-to-consumer options, there are a variety of ctDNA-based tests that can be performed. Each test has its own pros and cons, and their broad adoption necessitates rigorous evidence to support reliability, validity, cost-effectiveness and, most importantly, clinical utility – confirming that ctDNA-guided management can improve patient outcomes compared to the *status quo*^2^. Currently, insurance coverage or reimbursement from national health systems of ctDNA-based liquid biopsies in CRC is limited, only provided in the US and Japan, despite recommendations from leading international oncology societies for specific indications^1,3^. This discrepancy, combined with the rapidly growing number of publications on ctDNA, might lead to conflicting opinions among healthcare providers, as well as patients, about how and when ctDNA tests should be utilized in clinical care. Therefore, it is critical to distinguish between ctDNA applications that are well-supported by evidence and could be safely and effectively implemented in our clinical practice by tomorrow, and those that are still tentative and need further validation. As physicians and scientists, it is our responsibility to guide patients through the complexities of emerging technologies. Here we discuss emerging clinical applications of ctDNA tests in three main areas of CRC management (metastatic CRC, locally advanced disease and early disease), with the aim to ensure that expectations are realistic and informed by both the latest scientific evidence and individual patient contexts (also refer to Fig. 1).

### Reshaping the use of targeted therapies in metastatic disease with liquid biopsy

Metastatic CRC (mCRC) affects millions worldwide and is characterized by poor prognosis with a median overall survival of approximately 30 months, only 16% of long-term survivors and two-third of patients ineligible for second-line therapy due to rapid disease progression or poor performance status^4^. Additionally, the rapid development of resistance mechanisms (i.e., therapeutic-induced mutations) to both conventional chemotherapies and targeted therapies further complicates treatment efforts. These factors underscore the critical need for personalized, dynamic treatment strategies to improve patient outcomes^4^.

Liquid biopsy approaches have emerged as pivotal tools in guiding targeted treatment decisions for mCRC, that are based on the molecular profile of the tumor (i.e., actionable mutations that are targetable by a drug)^4^. Compared to invasive tissue biopsies, ctDNA analysis offers a safer and less invasive option. This can allow for serial monitoring of molecular changes in the cancer through routine blood draws, favoring timely and informed decisions^1^.

Proof-of-concept studies have supported the validity of ctDNA-guided treatment decisions in identifying therapeutic targets for patients with metastatic CRC who have exhausted standard-of-care therapy options. The utilization of ctDNA analysis has been effectively applied for treatment selection towards various mCRC targets, including *ERBB2* amplification, *KRAS*^G12C^ mutations, and EGFR signaling^5^.

For instance, repeated rounds of anti-EGFR therapies (known as anti-EGFR rechallenge) was previously empirical and is now transitioning to common practice due to ctDNA analysis^6^. While anti-EGFR drugs aim to inhibit mCRC cells, cancer may adapt, developing resistance mutations such as those in downstream oncogenes *KRAS*, *NRAS*, and *BRAF*. As sensitive cancer cells are reduced upon treatment, these mutant cells may continue to grow driving tumor progression. As different treatments are administered after anti-EGFR progression, resistant cells can be again cleared. This is where ctDNA analysis becomes essential. By monitoring genetic material from cancer cells in the blood, ctDNA analysis offers real-time insights into which mutations are present which helps oncologists determine when to reintroduce anti-EGFR therapies (specifically when resistant mutations are no longer detectable) to maximizes treatment efficacy^6^.

Additionally, FoundationOne Liquid CDx, a certified liquid biopsy test sequencing hundreds of cancer-associated genes, has recently gained FDA approval as a companion diagnostic test for prescribing encorafenib and cetuximab for mCRC patients with a *BRAF*^V600E^ mutation, and entrectinib for rare *NTRK1-3* fusions in solid tumors^7^. This development underscores the growing consensus that ctDNA holds significant promise as a precision medicine tool for mCRC in clinical practice.

To advance the use of ctDNA, the outcomes of phase III randomized control trials are eagerly awaited (Table 1a)^5^. However, to our knowledge, the LIBImAb trial (NCT04776655) is currently the only randomized trial that specifically investigates whether ctDNA can reliably guide the selection between anti-EGFR or anti-VEGF therapies (two critical classes of biological agents for mCRC care) alongside first-line chemotherapy. Additionally, research is moving towards other implementations, like using ctDNA for treatment monitoring to detect early molecular resistance mechanisms and possibly guide therapeutic switch before radiological progression^5^. Another potential use includes the investigation of blood TMB dynamics as a biomarker of response to immunotherapy for chemotherapy-primed mCRC^8^. It is important to note that ctDNA analysis is not yet fully developed for this wide variety of potential clinical applications beyond target identification in the chemorefractory setting, and substantiating its clinical advantages remains the primary objective of ongoing and future trials.

**Table 1:** Pivotal randomized trials investigating ctDNA-guided management in CRC (A) ctDNA-guided randomized trials in the metastatic setting, (B) ctDNA-guided randomized trials in the adjuvant setting

A							
Trial	Role of ctDNA	Phase	Therapy line	*N*	Arms	Primary endpoint	Est. duration
LIBImAb/ NCT04776655	ctDNA-guided selection between anti-EGFR or anti-VEGF therapies and therapeutic switch before radiological progression	III	First line	>300	Cetuximab + ChT vs Bevacizumab + ChT	PFS	2021–2024
NCT04509635	ctDNA-guided anti-EGFR rechallenge		Refractory	50	Cetuximab + ChT vs ChT alone	DCR	NA
MOLIMOR/ NCT04775862		II		>50	Anti-EGFR-based therapy vs regorafenib or FTD/TPI	ORR, PFS	2021–2024
PARERE/ NCT04787341				>200	Panitumumab vs regorafenib	OS	2020–2025
PULSE/ NCT03992456				>100	Panitumumab vs regorafenib or FTD/TPI	OS	2020–2024
CAVE-2 GOIM/ NCT05291156				>150	Cetuximab + avelumab vs cetuximab	OS	2022–2025
CITRIC/ EudraCT 2020-000443-31				>50	Cetuximab + irinotecan vs anti-EGFR free regimens	ORR	NA
Rapid 1/ NCT04786600	ctDNA-guided therapeutic switch before radiologic progression			>50	ctDNA-guided vs scan-guided intervention	OS	2022–2024
TACT-D/ NCT03844620				100	Regorafenib or FTD/TPI, ctDNA- vs scan-guided intervention	Early ctDNA changes, TRAE	2019–2026
1010(CG)2022-02/ NCT05815082	ctDNA-guided post-operative treatment (oligometastatic disease)	III	Resectable	>450	Watch-and-wait vs FOLFOX, if MRD- post-surgery	3-year PFS 5-year PFS	2023–2033
1010(PY)2022-10/ NCT05797077				>300	Capecitabine maintenance vs follow-up, if MRD+ after post-surgical FOLFOX/CAPOX		2023–2031
NCT04680260/ OPTIMISE		II		350	FOLFOXIRI if MRD+/De-escalation if MRD- vs SOC	2-year RFS	2021–2030

B							
Trial	Role of ctDNA	Phase	Stage	N	ctDNA assay method	Primary endpoint	Est. duration
SAGITTARIUS/ NCT06490536	De-escalation if MRD- and escalation if MRD+, including targeted therapy according to ctDNA-based molecular profile	III	II/III	700	Tissue-based NGS	2-year RFS	2024–2028
Circulate-US/ NCT05174169	De-escalation if MRD- and Escalation if MRD+			>1500		TTPos, DFS	2022–2030
DYNAMIC-III/ ACTRN12617001566325		II/III	III	1000		3-year DFS	NA
MIRROR/ NCT06204484		II	II/III	>300			2023–2028
TRACC/ NCT04050345	De-escalation if MRD-	III	II/III	1000	Blood-based ddPCR		2016–2031
VEGA/ JRCT1031200006	Escalation if MRD+			>1000	Tissue-based NGS		2020–2025
PRODIGE 70 – Circulate/ NCT04120701			III	>1500	Blood-based ddPCR		2020–2028
FINE/NCT05954078			II/III	>300	NA	ctDNA clearance	2023–2028
MEDOCC-CrEATE/ NCT06434896			II	>1300	Tissue-based NGS	Proportion of patients who accept AChT in MRD+	2020–NA
Circulate AIO-KRK-0217/ NCT04089631				>4500		DFS	2020–2026
AFFORD/ NCT05427669				>300		3‐year DFS	2022–2027
ERASE-CRC/ NCT05062889		II	II/III	>400		ctDNA clearance	2023–2027
Circulate-Spain/EudraCT 2021-000507-20				>100			NA
BNT122-01/ NCT04486378				>200	NA	DFS	2021–2027
IMPROVE-IT/ NCT03748680			I/II	64*	Tissue-based NGS		2024–2029
SU2C ACT3/ NCT03803553			III/IV	500	Blood-based NGS	DFS, ctDNA clearance	2020–2027
CIRCULATE-PAC PRODIGE 88/ NCT06197425	Escalation if persistent MRD+ after AChT	III	II/III	>1500	ddPCR	TTR	2024–2030
CLAUDIA/NCT05534087				>200	NA	3-year DFS	2022–2030
ALTAIR/ NCT04457297			III	>200	Tissue-based NGS	DFS	2020–2023
REVISE/ NCT06242418		II		60*	NA	Change in value of ctDNA concentration	2024–2026
NCI-2022-09129/ NCT05710406		II/III	II/III	>350	NA	DFS	2023–2034

*AChT* adjuvant chemotherapy, *ChT* chemotherapy, *CRC* colorectal cancer, *ctDNA* circulating tumor DNA, *DCR* disease control rate, *ddPCR* droplet digital PCR, *DFS* disease-free survival, *EGFR* epidermal growth factor receptor, *Est.* estimated, *FTD/TPI* trifluridine/tipiracil, *MRD* Minimal residual disease, *N* number of, *NGS* next-generation sequencing, *ORR* objective response rate, *OS* overall survival, *PFS* progression-free survival, *RFS* relapse-free survival, *TRAE* treatment-related adverse events, V*EGF* vascular endothelial growth factor, *TTPos* time to positivity, *TTR* Time-To-Recurrence.

### Liquid biopsy for locally advanced disease nears clinical implementation for minimal residual disease testing

Minimal residual disease (MRD) refers to cancer cells that can remain in the body after treatment and potentially lead to recurrence. Almost one third of patients with operable CRC recur after surgery, depending on tumor stage^9^. Nevertheless, clinical-radiological evaluation alone has no potential to accurately discriminate these cases. Detecting MRD is crucial because it helps predict the risk of cancer returning, informing further intervention to improve patient outcomes. The use of ctDNA analysis for detecting MRD after CRC surgery is nearing clinical application, particularly for guiding post-surgical treatment decisions^9^. In detail, it entails detecting ctDNA released by residual tumor cells (micrometastases) which may persist despite no radiological tumor evidence after radical surgery^9^. Detecting MRD differentiates patients at high risk of relapse from those who are probably cured by surgery alone, radically reshaping the current ‘*one-fits-all*’ standards of post-surgical care traditionally based on clinicopathological factors^9^. In this context, three key areas of application are emerging, each presenting unique goals and challenges concerning the sensitivity-specificity balance of ctDNA-based tests.

Firstly, for colon cancer (CC) stages where adjuvant chemotherapy is routinely administered based on clinical guidelines, namely high-risk stage II and stage III, ctDNA analysis offers the potential to identify individuals who might safely forego such treatment^9^. The randomized phase II DYNAMIC trial^10^ for locally invasive/node-negative disease (stage II) demonstrated that patients without detected ctDNA post-surgery could safely omit adjuvant chemotherapy without compromising relapse-free survival, with chemotherapy administered in 15% of cases including high-risk patients as compared to 28% in the standard-management group. The recently closed PEGASUS trial^11^ broadens the application of ctDNA to patients with locoregional disease (stage II high-risk and stage III). In this trial, the intensity of adjuvant chemotherapy in ctDNA-negative patients was reduced by eliminating the use of oxaliplatin, a highly neurotoxic drug, while ctDNA-positive patients received the standard 2-drug regimen CAPOX with oral fluoropyrimidine (CAPecitabine) and intravenous OXaliplatin. In these patients, additionally, PEGASUS evaluated the option of transitioning to an alternative chemotherapy regimen if ctDNA remained detectable despite treatment after 3 months of sequential monitoring. Notably, approximately 75% of participants in this trial tested MRD negative, underscoring the potential of ctDNA to spare a large number of individuals from unnecessary chemotherapy. It also showed that switching therapy can rescue patients whose MRD fails to respond to first-line CAPOX chemotherapy. The primary concern in these settings is the sensitivity of ctDNA assays rather than specificity, to avoid scenarios where patients requiring chemotherapy are falsely identified as ctDNA-negative. Further validation of these findings through larger randomized studies is ongoing (Table 1b)^9,12^. Development of novel ctDNA assay technologies may further enhance diagnostic performance.

Secondly, in stage II low-risk CC, most patients typically do not receive adjuvant chemotherapy due to low relapse rates (~10–15%). In this context, the emphasis is on identifying the few patients who truly require treatment, making specificity crucial to avoid overtreatment based on false-positive ctDNA results. The phase III COBRA trial^13^ randomized stage II low-risk CC patients to either standard-of-care surveillance or ctDNA-guided treatment with 6 months of oxaliplatin-based chemotherapy upon ctDNA positivity. Unfortunately, the trial was halted prematurely as it failed to reveal a significant difference in ctDNA clearance rates between the two arms. These findings highlight that the incorporation of ctDNA analysis in this particular subgroup needs further development and is still premature in clinical practice.

Lastly, in locally advanced rectal cancer (LARC), the AGITG DYNAMIC-Rectal trial^14^ utilized ctDNA to optimize adjuvant chemotherapy delivery, aligning with the approach seen in CC. The trial results not only emphasized ctDNA value as a prognostic indicator, but also showed that a ctDNA-guided approach to adjuvant therapy for LARC can reduce rates of chemotherapy administration (46% vs 76%). These results are now outdated due to evolving treatment strategies in LARC care^14^ as current treatment trends focus on intensifying pre-operative chemotherapy by utilizing Total Neoadjuvant Treatment (TNT) strategies. These include an oxaliplatin-based induction/consolidation regimen alongside the standard protocols of long-course chemoradiotherapy or short-course radiotherapy to reduce tumor size for less invasive surgical approaches. This shift, alongside COVID-related issues, unfortunately prompted the premature discontinuation of the DYNAMIC-Rectal trial, precluding conclusive findings^14^. Further research in LARC is now evolving towards elucidating ctDNA role in identifying patients who could avoid surgery following a clinical complete response to TNT^15^. ctDNA sensitivity is pivotal also in this setting to avoid false negative ctDNA results leading to undertreatment.

In conclusion, integrating ctDNA into clinical practice for locoregional disease comes with challenges, requiring standardized tests and tailored sensitivity/specificity based on tumor stage and treatment goals. Specialized centers can cautiously progress with ctDNA testing for patients with locoregional CC. As the field evolves, ongoing research and consensus-building are crucial for refining the role of ctDNA in treatment decisions across the remaining clinical settings. Despite these challenges, the results from over 10 ongoing RCTs in these contexts will soon provide valuable insights^12^.

### Early disease screening with liquid biopsy

The prevention of CRC hinges on effective screening campaigns including fecal occult blood tests (FOBT) and colonoscopy. However, participation rates in screening are well below 50% in many countries^16^, revealing a significant deficiency in early CRC detection. Non-invasive ctDNA testing has been proposed as a potential alternative. The ECLIPSE study, a significant research initiative involving over 7,500 patients at average risk for developing colorectal cancer (CRC), assessed the effectiveness of the ctDNA Shield test (Guardant Health) for early CRC detection in healthy individuals who are eligible for screening^16^. Overall, sensitivity and specificity for cancer detection were quite acceptable (83.1% and 89.6%, respectively). However, the ability to identify advanced precancerous lesions – the real gain of a secondary prevention program like CRC screening—was disappointingly only 13.2%, marginally above the false positive rate of 10.4% and well below the FOBT rate^16^. These figures indicate the limitations in ctDNA predictive value for these crucial conditions that necessitate effective screening interventions. Nevertheless, based on these results, the Guardant Health’s Shield ctDNA test was recently approved by the FDA as a primary screening option for CRC. It is our opinion that further research should rigorously address key issues like mortality reduction (effectiveness), method comparisons with other standard screening procedures like FOBT, adherence rates, economic impacts, optimal screening intervals, and improvement of sensitivity and specificity to mitigate false positives and unnecessary interventions^13^.

## Conclusions

In the evolving landscape of CRC management, ctDNA-based liquid biopsy presents the potential for a significant shift in refining treatment and care decisions. However, careful integration is necessary until comprehensive guidelines and conclusive results from ongoing randomized trials become available. It is important for both physicians and patients to recognize that, in the metastatic setting, while ctDNA tests can aid in the molecular selection of targeted therapies, their role in first-line treatments and other applications is still being researched. In the locoregional disease (resectable) setting, personalized strategies based on ctDNA-detected MRD are highly promising but still under evaluation. Thus, it is essential to wait for these results before fully incorporating ctDNA testing into treatment planning. Additionally, while the FDA has approved the first liquid biopsy test for cancer screening, limitations such as low sensitivity for detecting precancerous lesions must be considered to avoid false reassurance. This underscores the importance of not solely relying on this technology over other established diagnostic methods. Therefore, collaborative efforts among healthcare professionals, researchers, industry stakeholders, and patients are indispensable for crafting evidence-based approaches and protocols that will maximize the clinical utility of ctDNA tests and enhance patient outcomes. Presently, both physicians and patients should align their expectations with the current state of scientific evidence, tempering optimism with a clear understanding of ctDNA current limitations and future potential.
